# Oral Health Status and Factors Associated with Oral Health in Patients with Alzheimer’s Disease: A Matched Case-Control Observational Study

**DOI:** 10.3390/jcm14051412

**Published:** 2025-02-20

**Authors:** Reza Aghasizadeh Sherbaf, George Michael Kaposvári, Katalin Nagy, Magdolna Pakáski, Márió Gajdács, Danica Matusovits, Zoltán Baráth

**Affiliations:** 1 Department of Oral Surgery, Faculty of Dentistry, University of Szeged, Tisza Lajos krt. 64–66., 6720 Szeged, Hungary; aghasizadeh.reza@stoma.szote.u-szeged.hu (R.A.S.); kaposvari.george@stoma.szote.u-szeged.hu (G.M.K.); katalin.nagy@universityszeged.com (K.N.); matusovits.danica@stoma.u-szeged.hu (D.M.); 2 Department of Psychiatry, Faculty of Medicine, University of Szeged, Korányi fasor 8–10., 6720 Szeged, Hungary; babikne.pakaski.magdolna@med.u-szeged.hu; 3 Department of Oral Biology and Experimental Dental Research, Faculty of Dentistry, University of Szeged, Tisza Lajos krt. 64–66., 6720 Szeged, Hungary; gajdacs.mario@stoma.szote.u-szeged.hu; 4 Department of Prosthodontics, Faculty of Dentistry, University of Szeged, Tisza Lajos krt. 64–66., 6720 Szeged, Hungary

**Keywords:** dementia, Alzheimer’s disease, oral health, dental status, periodontal status, DMF-T, case-control study, epidemiology, gerodontology

## Abstract

**Background:** Alzheimer’s disease (AD) is a chronic neurodegenerative disease, ranking as the seventh leading cause of death in both sexes. There is increasing awareness of the role of chronic periodontal disease and severe tooth loss as a modifiable risk factor for developing AD. The aim of the present observational study was to assess AD patients with non-affected healthy controls in the context of their dental and periodontal health outcomes; additionally, the potential impact of anamnestic factors and lifestyle habits on oral health outcomes was also studied. **Methods:** A total of *n* = 41 AD patients receiving treatment at the Department of Psychiatry, University of Szeged, were compared with *n* = 41 age- and gender-matched controls from individuals seeking dental treatment and from retirement homes (mean age was 83.32 ± 7.82 years). Dental and periodontal status indices were assessed according to World Health Organization (WHO) criteria. **Results:** Overall, 51.2%, 68.3%, and 87.8% of AD patients received mood stabilizers, drugs for their non-cognitive symptoms and cognitive symptoms, respectively. Severe tooth loss was observed in 43.9% of AD patients and 56.1% of controls, respectively. There were no significant differences among AD patients and controls regarding the dental status indices studied (*p* > 0.05 for all indicators). AD patients had significantly higher plaque indices (%) (59.06 ± 15.45 vs. 41.35 ± 7.97; *p* < 0.001), bleeding on probing (BOP%) (62.65 ± 12.00 vs. 40.12 ± 10.86; *p* < 0.001), pocket depth [PD] (2.63 ± 0.56 vs. 2.29 ± 0.13; *p* = 0.002) and attachment loss [AL] (2.85 ± 0.79 vs. 2.39 ± 0.41; *p* = 0.026) values, compared to controls. Smoking (vs. non-smokers; 56.28 ± 12.36 vs. 51.40 ± 13.23, *p* = 0.038) and consumption of alcohol (vs. non-drinkers; 58.68 ± 9.86 vs. 54.78 ± 14.86, *p* = 0.040) were associated with higher plaque indices [%], while no similar effects were shown for dental status parameters (*p* > 0.05). In contrast, coffee intake and vitamin supplement use had no significant effect on dental or periodontal status parameters (*p* > 0.05 in all cases). **Conclusions:** The results of our study underscore the substantial treatment needs of AD patients, calling for heightened awareness among dental healthcare professionals.

## 1. Introduction

Degenerative diseases and pathologies with a delayed onset are expected to carry a rapidly increasing societal burden due to the late stages of the epidemiological transition, the substantial increase in life expectancy, and the aging of the global population [1,2]. According to recent projections, the share of the global population aged ≥65 years is expected to reach >16% by 2050 [3]. Of note, dementias—corresponding to a group of diseases with a heterogeneous pathophysiology, but similar clinical presentation—are noted as one of the most severe public health concerns of the 21st century [4,5]. Dementias include Alzheimer’s disease (AD; corresponding to 50–75% of all dementia diagnoses) and other forms of dementia, including vascular dementia, frontotemporal, Lewy body dementia, and mixed-form dementia [6,7]. Based on the estimates of the World Health Organization (WHO) for 2021, AD and other forms of dementia were responsible for the deaths of ~1.8 million people, ranking as the seventh leading cause of death in both sexes and ranking fourth in developed countries [8]. Furthermore, based on Global Burden of Disease (GBD) estimates, the prevalence of dementia is expected to rise from 57.4 million cases (2019) to 152.8 million cases by the year 2050 [9]; during the same time frame, dementia is projected to incur a cost of 14,513 billion international dollars (INT$, set at 2020 value), highlighting the sizable economic burden of disease, both for the caregivers and the healthcare systems [10].

The emergence of AD cases is bimodal: the overwhelming majority (>90%) of AD cases have an onset ≥65 years of age and are considered sporadic; on the other hand, <10% of cases present <65 years of age (“early-onset AD” usually between 30–60 years), where a there is a known familial history of the disease [11,12]. Based on disease severity, the illness may also be classified as mild, moderate, and severe AD [13]. AD leads to progressive changes in the central nervous system (CNS), which may be broadly described as the “AD continuum”: (a) in the first preclinical stage (which may last as long as 20 years), the biological onset of the illness and the various associated biomarkers may be detected, but the individual is symptom-free; (b) in the second stage, mild cognitive impairment may be noted by the individual or the close community around the individual (family members, friends), but these symptoms rarely interfere with the person’s ability to handle daily tasks; (c) the later stages of AD are characterized by severely impaired cognitive abilities and memory—limiting patients in executive functions, performing everyday tasks, and not recognizing loved ones—compromised ability to communicate, erratic (or often aggressive) behaviors and a tendency to wandering off [14,15]. Due to the nature of the disease, AD patients are characterized by substantial disability and dependency, leading to a significant decline in the quality of life (QoL) not only for AD patients but also for their families and caregivers [16]; in severe cases, appropriate treatment of these individuals may only be insured in long term care facilities [17]. At present, the most well-established theory for the etiopathogenesis of AD is associated with the deposition of β-amyloid plaques and neurofibrillary tangles, composed of hyperphosphorylated tau (τ) protein, resulting in elevated intracellular Ca^2+^-concentrations and an overall Ca^2+^-imbalance, consequently leading to the major loss of cholinergic neurons in the basal part of the forebrain [18,19,20]. Nevertheless, novel theories on the pathogenesis of AD, including alternative pathways of neuroinflammation or the role of oxidative stress, are emerging [21,22].

Among non-modifiable risk factors, advanced age, female sex and various apolipoprotein E (*ApoE*) and amyloid precursor protein (*APP*) alleles have been associated with developing AD, while metabolic syndrome (i.e., hypertension, high fasting plasma glucose, elevated cholesterol, obesity) and associated atherosclerosis, inadequate physical activity, excessive alcohol consumption, hearing loss and other co-morbidities (e.g., depression) are among the modifiable risk factors for developing the disease [23,24]. The interplay between oral health and AD (and dementia more broadly) has been the subject of substantial attention [25]; on one hand, with the progression of the disease, AD patients are increasingly limited in performing everyday tasks, such as keeping up appropriate oral hygiene practices (which may be compounded by the loss sensory and motor functions associated with physiological aging), leading to substandard oral health outcomes and oral health-related QoL [26]. Furthermore, various medicines administered as adjunctive treatment (e.g., mood stabilizers, benzodiazepines, antipsychotics), or to target the cognitive symptoms of the patient may present with severe adverse events in the oral cavity, further exacerbating AD patients’ oral health status [27,28]. Thus, the upkeep of AD is a crucial domain within the scope of gerodontology.

In recent years, numerous publications have highlighted the correlation between oral health and the development of systemic illnesses; for example, periodontitis has been demonstrated as a major risk factor for coronary heart disease and/or myocardial infarction [29,30] and diabetes [31], respectively. In the same vein, there is increasing awareness about the role of oral health as a risk factor for developing AD and other types of dementia; the link between severe tooth loss and edentulism and cognitive impairment has been established by numerous epidemiological studies [32,33]. Additionally, the chronic and systemic inflammation caused by the dysbiosis in the oral cavity due to the progression of periodontal disease has also been associated with the pathogenesis of AD-associated cognitive impairment, and other neurodegenerative diseases [34]. Among periodontopathogenic bacteria, the role of *Porphyromonas gingivalis* (*P. gingivalis*)—and its virulence factors—is the most well-established, which are capable of passing through the blood–brain barrier (BBB), contributing to direct neuronal damage, neuroinflammation and β-amyloid plaque formation [35]. Furthermore, a recent population-based data linkage study by the National Institute of Aging (NIA)—based on the National Health and Nutrition Examination Survey (NHANES) population—has shown a notable association with diagnoses of AD or other dementia and AD-associated deaths and antibodies against *P. gingivalis.* Moreover, the study found that the mortality risk increased if results were clustered with antibodies against other oral bacteria (including *Campylobacter rectus* and *Prevotella melaninogenica*), highlighting the role of bacteria implicated in periodontal disease in the pathogenesis of AD and cognitive decline [36]. Thus, health policy initiatives should focus on the delivery of the appropriate dental healthcare services as needed, initially, to reduce the risk and extent of chronic inflammatory processes via preventive interventions, furthermore to ensure the best possible oral health outcomes and oral health-related QoL in patients affected by AD.

Although numerous epidemiological studies have assessed the dental and periodontal indicators of AD patients alone [37,38], comparative analyses of their oral health status with members of the general population are scarce. Hence, the purpose of the present observational study was to analyze AD patients with non-affected healthy controls in the context of their dental and periodontal health outcomes. Furthermore, the possible role of underlying patient characteristics and lifestyle patterns on oral health outcomes of AD patients was also assessed. We have determined the following initial hypotheses: (a) the dental status indices of AD patients are worse than in healthy control participants, (b) the periodontal status indices of AD patients are worse than in healthy control participants, (c) dental and periodontal status parameters do not show differences on the basis of the participant’s sex, (d) lifestyle factors (i.e., tobacco consumption, alcohol use, coffee intake, vitamin supplement use) affect the oral health status parameters assessed.

## 2. Materials and Methods

### 2.1. Study Design, Timeline

The present research was based on an observational, matched case-control study design. An expected sample size of *n* = 40 individuals per study group (cases and controls, respectively) was determined for a study of inter-observer agreement with two raters, with a statistical power of 80% and α set at 5% [39]. The present study was carried out between 1 November 2016 and 31 December 2021.

### 2.2. Study Participants, Study Setting

Participants of the study consisted of the following two populations: cases, i.e., individuals diagnosed with AD, receiving treatment as outpatients or inpatients at the Department of Psychiatry (DoP), Albert Szent-Györgyi Health Centre (HC), University of Szeged (USz), Hungary. The HC is a tertiary-care teaching hospital situated in the Southern Great Plain of Hungary. The clinical diagnosis of AD was confirmed by initial evaluation through comprehensive history taking (including personal and family history), neurological and psychiatric examinations, together with assessment via psychometric tests to confirm cognitive impairment [40]. Cognitive assessment of AD patients was performed using a short battery of neuropsychological tests, including the Mini-Mental State Examination (MMSE), while the depressive symptoms were screened using the Geriatric Depression Scale (GDS) [38,41]. Furthermore, brain computer tomography (CT) or magnetic resonance imaging (MRI) scan was conducted for each individual. A routine laboratory workup, including thyroid function tests (TFT), was also carried out [42]. All eligible participants fulfilled the criteria outlined in the Diagnostic and Statistical Manual of Mental Disorders, 5th Edition (DSM-5), and had probable AD, according to the criteria of the National Institute of Neurological and Communicative Disorders and Strokes—AD and Related Disorders Association (NINCDS–ADRDA) [43,44]. A random stratified sampling design—taking into consideration age and gender—was used to select patients with AD (ranging from mild to severe cases) from an overall cohort of patients treated at the DoP [45].

The second population in our study were controls, i.e., individuals who were selected from a generally healthy, cognitively normal cohort willing to participate, without neurodegenerative or psychiatric disorders (or taking any medication related to these disorders) or other severe systemic illnesses. Participants were originating from (a) individuals requiring dental treatment at the Faculty of Dentistry, USz; (b) retirement homes situated in Kistelek (~30 km away from the HC) and Szentlőrinc (~170 km away from the HC). Participants in the case and control groups were matched according to their age (±3 years) and gender distributions to allow for more reliable comparisons in our analysis [46].

### 2.3. Determination of Oral Health Status, Clinical Procedures

The oral health status assessment in AD patients (cases) and control subjects was carried out through an exhaustive full-mouth dental and periodontal status assessment based on the World Health Organization (WHO) criteria; the practical aspects of the oral health examinations and the dental and periodontal indices recorded were described in detail elsewhere [47,48,49]. Edentulous cases and their corresponding controls were not included in the study of periodontal status parameters [47]. The oral health status assessment of AD patients was carried out under the supervision of a psychiatric care physician at all times. During the examination of the patients, the oral cavity was illuminated with a penlight and evaluated visually using a dental mirror and tweezers. The clinical examination also included using periodontal probes and panoramic radiographs (OPG). All oral health assessments were carried out by two dentists (G.M.K. and R.A.S.; each having over five years of clinical experience), to ensure consistency in these examinations. Following oral health assessments, instructions on appropriate were given by the operators.

### 2.4. Data Collection on Anamnestic Data and Lifestyle Factors

To complement the data collected during the oral health assessments, data on underlying patient characteristics and lifestyle patterns has also been gathered, corresponding to factors that could impact the studied oral health outcomes [47]. The following information was collected through direct questions from the examiners: (a) sex, (b) age, (c) tobacco consumption (expressed as cigarettes/day), (d) alcohol use (expressed as units/week), (e) coffee intake (expressed as cups of coffee/day), (f) consumption of vitamin supplements (the individual takes vitamin supplements/the individual does not take supplements) [47], (g) use of mood stabilizer medications (i.e., citalopram and duloxetine), (h) use of drugs for the treatment of the non-cognitive symptoms (behavioral and psychological, i.e., tiapride and risperidone), and (i) use of drugs for the treatment of cognitive symptoms (i.e., donepezil, rivastigmine, piracetam, and nicergoline).

### 2.5. Statistical Analysis

During descriptive statistical analysis, categorical variables were shown as frequencies and percentages (*n*, %), while continuous (numerical) variables were described as means and standard deviations (mean ± SD). The normal distribution of the dataset was assessed using the graphical method (quantile–quantile plot) and Shapiro–Wilk tests. The following univariate analyses were performed: (a) to detect differences between proportions, the χ^2^-test and Fisher-exact tests were used (with a Cramér’s phi (φ) effect size measure); (b) comparisons between groups in regards to continuous data were performed using Mann–Whitney U-tests. Inferential analyses were carried out using SPSS Statistics version 26.0 (IBM Inc., Chicago, IL, USA); *p* values below 0.05 (*p* < 0.05) were considered statistically significant.

### 2.6. Ethical Considerations

The present study was carried out in accordance with the Declaration of Helsinki (1975, last revised in 2013) and national and institutional ethical standards. Ethical approval for the study was obtained from the Human Institutional and Regional Biomedical Research Ethics Committee, University of Szeged (SZTE-RKEB), Hungary (reference number: 170/2016-SZTE [3867]; ethical approval date: 17 October 2016) and the Hungarian Medical Research Council (ETT-TUKEB; reference number: IV/2426-2/2020/EKU; ethical approval date: 1 April 2020). Written informed consent was obtained from all the participants involved in this study or their legal guardians. They were briefed about the research objectives, privacy, and confidentiality of their data, and they were made aware that their participation in the research was voluntary and that they may withdraw from the study at any time. During data collection, the anonymity of the patients and controls was preserved.

## 3. Results

### 3.1. Demographic Characteristics, Anamestic Data

Forty-one (*n* = 41) patients with AD (i.e., cases) were included in the study, along with forty-one (*n* = 41) accurately matched control subjects without AD; a summary of demographic characteristics, anamnestic and lifestyle factors of patients with AD and controls is presented in Table 1. The demographic characteristics of the subjects were the following: (a) gender: 17 male (41.5%) and 24 female (58.5%), (b) age: the mean age was 83.32 ± 7.82 years (range: 66–97), with the following distribution: 66–77 years *n* = 11 (26.8%), 78–87 years *n* = 17 (41.5%), 88–97 years *n* = 13 (31.7%) (Table 1). On the basis of age or gender, no significant differences were present between the two groups (*p* = 1.000 in both cases). No significant disparities were noted in relation to tobacco use (*p* = 0.676, φ = 0.097), alcohol consumption (*p* = 0.956, φ = 0.03), coffee consumption (*p* = 0.156, φ = 0.173), and the reported use of vitamin supplements (*p* = 0.085, φ = 0.19), respectively. 56.1% and 46.3% of patients with AD and controls were non-smokers, respectively. At the time of the study, 51.2% (*n* = 21), 68.3% (*n* = 28), and 87.8% (*n* = 36) of patients received mood stabilizers, drugs for their non-cognitive symptoms and cognitive symptoms, respectively. For the purposes of inferential statistics, the following dichotomous subgroups were made: (a) smoking: non-smoker (0 cigarettes/day) vs. smoker (≥1 cigarettes/day), (b) alcohol use: non-drinker (0 units/week) vs. drinker (≥1 units/week), (c) coffee intake: 0–1 cups/day vs. ≥2 cups/day, (d) vitamin supplements: taking them vs. not taking them.

### 3.2. Dental Status Parameters in AD Patients and Controls

Dental status indicators corresponding to AD patients and controls are summarized in Table 2. There were no significant differences between AD patients and controls corresponding to DMF-S (119.83 ± 16.78 vs. 119.00 ± 18.93; *p* = 0.563), D (4.56 ± 4.18 vs. 4.02 ± 4.95; *p* = 0.599), M (20.66 ± 7.29 vs. 21.37 ± 7.76; *p* = 0.672), F (1.02 ± 2.23 vs. 0.78 ± 1.82; *p* = 0.590), DMF-T values (26.24 ± 3.58 vs. 26.15 ± 4.29; *p* = 0.911), and the number of crowns (3.61 ± 5.23 vs. 3.76 ± 5.51; *p* = 0.903), respectively (Table 2). Severe tooth loss was observed in the case of *n* = 18 (43.9%) of patients with AD and *n* = 23 (56.1%) of controls. In the context of gender, no significant differences were observed among male vs. female subjects with regards to D (3.83 ± 4.45 vs. 4.94 ± 4.74; *p* = 0.284) and F (0.67 ± 1.75 vs. 1.24 ± 2.36; *p* = 0.314) values and the number of crowns (3.23 ± 4.99 vs. 4.32 ± 5.94; *p* = 0.314), while a numerical tendency was seen for M (22.42 ± 6.43 vs. 19.03 ± 8.47; *p* = 0.054), DMF-T (26.87 ± 2.74 vs. 25.21 ± 4.74; *p* = 0.064) and DMF-S (122.46 ± 13.09 vs. 115.12 ± 22.36; *p* = 0.065) values, which consistently showed higher values for females. Among female subjects, no significant differences were shown between patients with AD and controls, corresponding to DMF-S (121.21 ± 15.96 vs. 123.71 ± 9.61; *p* = 0.549), D (4.67 ± 4.65 vs. 3.00 ± 4.48; *p* = 0.198), M (21.45 ± 7.05 vs. 23.38 ± 5.74; *p* = 0.307), F (0.38 ± 1.50 vs. 0.95 ± 2.23; *p* = 0.251), DMF-T values (26.50 ± 3.26 vs. 27.29 ± 2.12; *p* = 0.323) and the number of crowns (2.79 ± 4.71 vs. 3.66 ± 5.33; *p* = 0.514), respectively (Table 2). Similarly, among male subjects, no significant differences were shown between patients with AD and controls corresponding to DMF-S (117.88 ± 18.19 vs. 112.35 ± 26.10; *p* = 0.479), D (4.41 ± 3.59 vs. 5.47 ± 5.74; *p* = 0.533), M (19.53 ± 7.69 vs. 18.53 ± 9.41; *p* = 0.737) and DMF-T values (25.88 ± 4.08 vs. 24.53 ± 5.91; *p* = 0.443) and the number of crowns (4.76 ± 5.91 vs. 3.88 ± 6.13; *p* = 0.672), respectively; on the other hand, numerical tendency was seen in the case of F values (1.94 ± 3.05 vs. 0.52 ± 1.06; *p* = 0.081), with higher values seen in AD patients. Based on smoking habits (non-smoker vs. smoker), alcohol use (non-drinker vs. drinker), coffee intake (0–1 cups/day vs. ≥2 cups/day), and vitamin supplement use (the individual takes vitamin supplements vs. the individual does not take supplements), no significant differences were seen for any of the dental status parameters studied (*p* > 0.05 in all cases).

### 3.3. Periodontal Status Parameters in AD Patients and Controls

Periodontal status indicators corresponding to AD patients and controls are summarized in Table 3. AD patients had significantly higher plaque indices (59.06 ± 15.45 vs. 41.35 ± 7.97; *p* < 0.001), BOP% (62.65 ± 12.00 vs. 40.12 ± 10.86; *p* < 0.001), PD (2.63 ± 0.56 vs. 2.29 ± 0.13; *p* = 0.002) and AL (2.85 ± 0.79 vs. 2.39 ± 0.41; *p* = 0.026) values, compared to controls (Table 3). Similarly, when comparing male (plaque indices: 58.44 ± 17.39 vs. 43.22 ± 9.23, *p* < 0.001; BOP%: 65.44 ± 12.70 vs. 40.33 ± 13.22, *p* < 0.001; PD: 2.60 ± 0.73 vs. 2.27 ± 0.12, *p* = 0.0015; AL: 2.73 ± 0.97 vs. 2.24 ± 0.73, *p* < 0.001) and female (plaque indices: 59.75 ± 14.11 vs. 39.25 ± 6.21, *p* < 0.001; BOP%: 59.50 ± 11.11 vs. 39.88 ± 8.42, *p* < 0.001; PD: 2.68 ± 0.32 vs. 2.32 ± 0.15, *p* < 0,001; AL: 2.98 ± 0.53 vs. 2.53 ± 0.46; *p* = 0.015) AD patients and control subjects separately, significant differences were highlighted (Table 3). In the context of gender, no significant differences were observed among female and male subjects regarding plaque index (49.55 ± 13.90 vs. 50.83 ± 15.68; *p* = 0.246), BOP% (49.69 ± 13.90 vs. 52.89 ± 18.03; *p* = 0.303), PD (2.50 ± 0.31 vs. 2.43 ± 0.54; *p* = 0.378) and AL (3.03 ± 0.98 vs. 2.63 ± 0.75; *p* = 0.375) values, respectively. Based on smoking habits (non-smoker vs. smoker), plaque indices were significantly higher in smokers (51.40 ± 13.23 vs. 56.28 ± 12.36, *p* = 0.038), while in case of BOP% (52.87 ± 13.65 vs. 53.76 ± 11.40; *p* = 0.200), PD (2.45 ± 0.37 vs. 2.56 ± 0.55; *p* = 0.423) and AL (2.61 ± 0.41 vs. 2.71 ± 0.84; *p* = 0.370) values, no significant differences were seen. Based on alcohol consumption (non-drinker vs. drinker), plaque indices were significantly higher in individuals who consumed alcohol (54.78 ± 14.86 vs. 58.68 ± 9.86, *p* = 0.040), while in case of BOP% (53.76 ± 21.40 vs. 55.38 ± 11.58; *p* = 0.254), PD (2.46 ± 0.53 vs. 2.55 ± 0.34; *p* = 0.567) and AL (2.69 ± 0.81 vs. 2.62 ± 0.39; *p* = 0.502) values, no significant differences were seen. In contrast, according to coffee intake (0–1 cups/day vs. ≥2 cups/day) and vitamin supplement use (the individual takes vitamin supplements vs. the individual does not take supplements), no significant differences were seen for any of the periodontal status indices studied (*p* > 0.05 in all cases).

## 4. Discussion

The aging of the global population is accelerating, with estimates from the United Nations (UN) projecting the elderly population to double by 2050 [50]; these demographic changes are causing particular challenges for healthcare systems (costs of medical care) and for societies (social care costs) alike [3,51]. For this purpose, the UN has announced the “The Decade of Healthy Aging” program (2021–2030), with the aim of improving the lives of the elderly and their caregivers, maintaining their health and dignity, and ensuring sustainability on a global scale [52]. Patients with dementias—among which AD is the most common—will have difficulties in performing activities of daily living, while in the late stages of the disease, they may require intensive, around-the-clock care for several years [53]. As the therapeutic options of AD—which are mostly limited to the management of symptoms—are limited, all interventions targeting the reduction of AD-related disease burden are critical, both from an epidemiological and economic context [54]. In addition to age, many lifestyle factors and co-morbidities are associated with the increased risk of developing AD later in life [16,23,55]; of note, the relationship between oral health and AD has received considerable attention. Thus, health policy considerations should strengthen the initiatives in support of integrating oral healthcare for aging populations, both from the context of retained functionality and autonomy, and to reduce an important modifiable risk factor for AD [56].

There is increasing evidence to support the bidirectional relationship between oral health and cognitive function, with severe tooth loss, edentulism, and a decline in masticatory function—in a similar fashion to hearing loss and vision impairment—leading to a compounding loss in self-sufficiency in elderly people [57,58]. In a meta-analysis of longitudinal studies by Qi et al., a dose–response relationship was identified between tooth loss, cognitive impairment (1.48 times higher risk), and a diagnosis of dementia (1.28 times higher risk), even after controlling for other variables [59]; on the other hand, the association was not significant in individuals using dentures. This protective effect of denture use against the rate of cognitive decline was also demonstrated by other studies [60]. Furthermore, numerous studies underpin the relationship between periodontal disease and its clinical consequences (i.e., chronic inflammation, AL, increased PD, and alveolar bone loss) and the pathomechanism of AD [61]; as the global prevalence of periodontitis is 30–50%–out of which ~19% of the world’s population is affected by severe chronic periodontal disease—this association carries considerable epidemiological relevance [62]. The oral microbiota consists of >700 distinct bacterial species, out of which *P. gingivalis* has the most pronounced role in maintaining the chronic and destructive inflammatory processes leading to periodontal disease [34,63]. This pathogen has the ability to pass on through the BBB via the bloodstream and possesses various virulence factors (i.e., lipopolysaccharide, gingipain, capsule, fimbriae, proteases for the cleavage of anti-inflammatory cytokines and outer membrane vesicles (OMVs)) to maintain a persistent, dysregulated, systemic inflammatory cascade [64,65]. The systematic review of Said-Sadier et al. performed the narrative synthesis of studies on the association between periodontitis and cognitive impairment/dementia and AD pathology; their summary revealed two distinct but interconnected mechanisms by which *P. gingivalis* contributes to dementia risk [66]. Direct effects include the invasion of the brain by the pathogen via the BBB, impairing the function of nerve cells and glial cells by direct neurotoxicity (through gingipains secreted by the bacteria), neuroinflammation, and contributing to plaque formation [64,65,66]. Other studies also highlighted that gingipains (i.e., cysteine proteases) may also have a role by increasing the permeability of human cerebral microvascular endothelial cells—by affecting tight junctions—facilitating invasion [64,67]. Indirect effects include the release of pro-inflammatory (IL-1, IL-6, and TNF-α) cytokines and reduction of anti-inflammatory mediators (e.g., epidermal growth factor, interferon-induced protein 10 (CXCL10), monocyte chemoattractant protein-1) leading to a pathological immune response [64,65,66]. Based on the narrative synthesis, the risk for dementia is higher in individuals affected by chronic periodontitis for ≥8 years [66].

In the present single-center study, the dental and periodontal status of forty-one AD patients were surveyed and subsequently compared to cognitively healthy, age and sex-matched controls; our study aims to highlight the dental healthcare burden of this population with special dental care needs and to showcase disparities in oral disease burden between physiological aging and AD. The study used standard methodology to assess dental and periodontal health and mental status (the MMSE was the most commonly used test in the published literature to determine cognitive status [66]) and had a similar sample size and duration with other reports to allow for comparison. In contrast to our initial hypothesis, no significant differences were shown among AD patients and the controls in our sample regarding the main caries status indicators (i.e., DMF-T and DMF-S) assessed, and the number of crowns (which was considered a proxy measure of an individual’s health-seeking behavior). D, M, and F teeth or the presence of crowns (70.7% vs. 53.7%, 100.0% vs. 100.0%, 29.3% vs. 24.4%, and 31.7% vs. 41.5%, respectively) were prevalent in both the AD patients’ group and among controls. Our results are in agreement with the findings of a systematic review by Delwel et al., where the dental status of older people with and without dementia were compared [68]. Their summary found no relevant differences overall—highlighting only one study, where dementia patients had significantly worse DMF-T and oral health index (OHI) values compared to healthy individuals [69]—while noting that root caries was more common in dementia. The M and DMF-T values from our participants were on the higher end (M_without dementia_: 19.7–26.1, M_with dementia_: 14.9–28.0, DMF-T_without dementia_: 10.2–27.3, and DMF-T_with dementia_: 9.3–28.2) of values reported previously for both groups (our results being more homogeneous to studies from developing countries), especially for M teeth, even though the majority were in a similar age range [68]. Although a tendency for higher M values was shown in females, differentiation in dental status according to sex was not seen; likewise, the majority of related studies did not find or did not report sex-based differences in dental status [68], with the exception of one study based in nursing homes, where significantly more males were dentate [70]. In addition to advanced age, the strategies applied in the dental care of patients with dementia may explain the high number of M teeth and DMF-T values in our study and in the published literature [68]; in many instances, tooth-conserving methods (which are more labor-intensive, and would require additional follow-ups and the adherence of the patients) are waived in exchange for the strategy to extract broken/decayed teeth, due to the challenges in facilitating the effective dental treatment of AD patients [32].

In line with our initial hypothesis, patients affected by AD showed significantly worse periodontal parameters (plaque index, BOP%, PD, and AL) in comparison with control subjects; this underscores the findings in the systematic review and meta-analysis by Maldonado et al., where the clinical periodontal status of older people with our without dementia was compared, collecting data on the same indicators as used in our study (i.e., plaque index, PD, BOP%, clinical AL and bleeding index) [71]. Quantitative analysis revealed a weighted mean difference of 35.72% (~22% in our study), 6.98%, 15.95% (~18% in our study), 2.53 mm, and 1.46 mm (~0.4 mm in our study) for BOP%, bleeding index, plaque index, clinical AL and PD, respectively. Compared to the above results, differences in periodontal status indicators between cases and controls were less pronounced in our sample, which may explained by differences in the diagnostic criteria used and the progression of the disease (mild vs. severe, which would inherently affect the executive functions of AD patients), the heterogeneity in the AD patients’ age (ranging from 50–107 years), differences in sample sizes (ranging between 52–409), or the overall worse oral health condition of our controls [71]. Participants did not show relevant differences in their periodontal status parameters on the basis of gender, which also confirmed our working hypothesis, while one study showed significantly higher values for inflammatory markers (e.g., leukocytes, neutrophil counts, C-reactive protein) in male AD patients [72], neither this nor other related studies showed sex-based differences in the periodontal status indicators assessed. The systematic reviews of Delwel et al. [68] and Maldonado et al. [71] also showcased the limited number of studies on AD patients with a case-control study design from Central and Eastern European countries.

The study also assessed the potential effects of various lifestyle factors—which were well-matched among AD cases and controls—on oral health parameters: overall, smokers (vs. non-smokers) and individuals consuming alcohol (vs. non-drinkers) had comparatively worse plaque indices (%), but similar differences were not shown for other periodontal indicators. Similarly, neither of the lifestyle factors had relevant effects on caries experience in our sample. Our findings correspond to the results of Panzarella et al., involving patients with AD and mild cognitive impairment [73]; while male sex and an AD diagnosis were associated with worse DMF-T indices and a higher microbial load of relevant periodontopathogens, similar differences in the context of smoking were not shown. Furthermore, the study of Calzada et al.—involving cognitively healthy individuals aged ≥60 years old—did not confirm smoking as a significant predictor for mandibular edentulism [74]. Likewise, Holmer et al. showed—in a comparative study with a sample of individuals with AD, varying levels of cognitive decline, and healthy individuals—that while AD patients had significantly higher rates of deep periodontal pockets and BOP, differences in smoking habits (i.e., current–previous–never) did not affect clinical parameters and microbial alpha-diversity. Their results underlined that specific shifts in microbial composition, characteristic of periodontal disease, were observed in the subgingival microbiota of AD patients [75]. Tobacco consumption may also contribute to the progression of AD via the nexus of the oral microbiota by leading to a shift towards high-risk periodontopathogenic bacteria in the oral cavity and by increasing the expression of bacterial virulence factors. Furthermore, components of cigarette smoke impair the function of microvascular endothelial cells and damage tight junction proteins (facilitating the invasion of oral bacteria through systemic circulation), and they increase the risk of plaque deposition in the brain [76]. The evidence for alcohol consumption is an independent risk factor for periodontal disease (by being a direct irritant to gingival tissues, affecting the immune response, leading to higher inflammatory marker levels, and affecting decision-making and nutrition) is well-established and is characterized by a linear dose–response relationship [77]. The meta-analysis of Yussof et al. also highlighted that alcohol consumption (focusing on binge drinking) contributes to the pathogenesis of AD via multiple pathways, inducing various signaling mechanisms and dysbiosis in the oral microbiota, altering BBB permeability [78].

Dental and periodontal health may also be markedly influenced by qualitative and quantitative aspects of one’s nutrition, especially in the context of elderly patients [55,79]. On the one hand, a lack of appetite, reduced olfactory and gustatory capacities, a monotonous diet, severe limitations in activities of daily functioning, or unnoticed orofacial pain may lead to various nutritional deficiencies—such as protein-energy malnutrition, lack of minerals, vitamins–carrying considerable risks. The sufficient intake of vitamin C (acting as a co-factor for collagen synthesis, ensuring the appropriate functioning of the gingiva and periodontal ligaments) and D (described as having anti-inflammatory properties and essential to regulating the mineral density of teeth) is especially important for AD patients [80]. In addition, the frequent consumption of foodstuffs (e.g., fruit juices and coffee) containing large amounts of acids may lead to dental erosion [81]. Our study assessed whether coffee consumption and/or vitamin supplement use had any effect on oral health parameters, which showed no significant differences. In a data-linkage study using the “BigMouth” dental data repository, Saleh et al. collected data on the consumption of 21 vitamins and supplements and periodontal health indicators: with the exception of iron supplements and multivitamins, the users of the majority of products did not observe significant benefits [82]. A systematic review and meta-analysis by Rhee et al., based on available studies, has failed to show a significant association between coffee consumption and periodontal diseases [83]. These observations were confirmed by Liao et al., whose study employed a Mendelian randomization design, which also pointed to the lack of a relationship between coffee-consuming behavior and the onset of periodontitis [84]. On the other hand, the hospital-based case-control study of Hou et al. has shown the protective effects of consuming ≥3 cups of coffee or tea per day against AD and vascular dementia, respectively; their study has also highlighted that this protective effect was more pronounced for hypertensive individuals and female patients [85].

From the standpoint of oral healthcare, elderly individuals represent a vulnerable patient population with special treatment needs [86]; barriers to treatment and/or preventive services are further compounded in the case of individuals affected by dementia. AD leads to the progressive loss of cognitive functions and autonomy, with limited therapeutic options available at present. The aim of pharmacological treatments is to postpone or slow down the decline in cognitive function, to address the behavioral aspects of the disease, and to treat associated co-morbidities; these drugs are prescribed to ensure the best possible QoL for AD patients and to decrease the burden for their caregivers [7,48]. Medicines used to treat and modulate cognitive symptoms include cholinesterase inhibitors (donepezil, rivastigmine, galantamine, and tacrine), the N-methyl-D-aspartate (NMDA) receptor antagonist memantine, and nootropics (such as piracetam and nicergoline). Furthermore, additional drugs may be prescribed adjunctively to treat sleep disturbances (e.g., benzodiazepines and zolpidem), agitation with or without psychosis and delusions (e.g., risperidone, quetiapine, and tiapride), depression (e.g., selective serotonin reuptake inhibitors (SSRIs) and serotonin-noradrenaline reuptake inhibitors (SNRIs)) and apathy (e.g., methylphenidate) [15,22,23,27,28,87]. Over half of the AD patients in our study received mood stabilizers, while the majority were on neuroleptics and cholinesterase inhibitors, respectively. Adverse effects of the above drugs—especially in relation to adjunctive treatments—may lead to xerostomia, involuntary movements, and a net decline in the density of beneficial bacteria in the oral cavity, further impairing the physiological functions of the mouth [88]. Recent advances in AD pharmacology shifted towards targeting the pathomechanisms and progression of the disease: to this end, various monoclonal antibodies (i.e., aducanumab, lecanemab, and donanemab) have been studied, which act by inhibiting the formation of senile plaque via targeting APP [23,54,89]. However, the developments in this field were not without their challenges: the commercialization of aducanumab has been recently discontinued due to insufficient evidence of its efficacy and frequent reports of notable adverse events (amyloid-related imaging abnormalities with edema or effusions) following an intensely scrutinized accelerated approval decision by the Food and Drug Administration [23,54,89,90].

As effective therapeutic alternatives for AD are still scarce, the societal value of providing preventive dental care services and controlling the burden of periodontitis, severe tooth loss, and edentulism—being significant risk factors for its development—should not be underestimated in reducing the global dementia burden [9,52]. Furthermore, as AD patients often receive suboptimal dental healthcare, tailored approaches should be facilitated to maintain oral functionality, autonomy, and dignity for as long as possible, which will also result in positive add-on effects for their families and caregivers [52,91]. As the disease progresses towards its moderate and severe stages, the upkeep or personal oral hygiene habits may become challenging to individuals: they may forget the goal and specific steps (e.g., toothbrushing with toothpaste, rinsing, flossing) of their oral hygiene routines, or have difficulties in performing them due to tremors or loss of motor coordination [26]. Motor and non-motor symptoms of AD will inevitably lead to significant impairment, affecting ordinary gestures and the individual’s personal oral hygiene practices [92]. In the case of patients wearing dentures, their appropriate upkeep and cleaning practices may also be neglected. Oral health often becomes less of a priority in case of patients requiring 24/7 care by their family or caregivers (as they are struggling with more urgent everyday concerns), in nursing homes (nursing staff and allied healthcare professionals are not qualified to perform it) or for patients institutionalized due to their behavioral symptoms or delusions (oral care is often not provided) [35,87]. The role of dental hygienists as qualified healthcare professionals is to administer competent and sensitive oral healthcare, in addition to supporting their physical and emotional well-being through flexibility and continuous follow-up [93]. The review of Marchini et al. identified five levels of barriers limiting oral healthcare in AD patients: *(a) personal level*, i.e., the decline in executive function, resistance to assistance from others, aggression, and impaired ability to provide informed consent; *(b) population/societal level*, i.e., there are not enough dental healthcare professionals available for the growing number of patients with dementia; *(c) professional level*, i.e., many dental healthcare professionals do not feel comfortable or competent to give treatment to dementia patients or to work in long-term care facilities; *(d) institutional level*, i.e., in many settings (e.g., nursing homes), they are unable to provide appropriate oral healthcare; *(e) healthcare system level*, i.e., dental care is not included in the country’s health insurance schemes, or limited reimbursement rates associated with domiciliary care [94].

Limitations of the present study should be acknowledged, including the relatively small sample size and its single-center nature; furthermore, the results may also be affected by selection bias, as cases in our study were taken from a pool of patients treated at a tertiary-care clinical center offering specialized facilities. Thus, the above points may affect the external validity and generalizability of our findings for other settings. Furthermore, information on various socio-demographic characteristics (e.g., living standards, socio-economic stratification, highest educational level, and exposures associated with occupational hazards), lifestyle characteristics and risk-taking behaviors, and co-morbidities—which could affect outcomes—either in relation to AD disease stage and/or oral health—were not comprehensively assessed for our participants. Furthermore, we did not possess information on the personal oral hygiene habits of the patients preceding their involvement in the study. On the other hand, the characteristics of our case-control study, the diagnostic criteria, and the methods used during the assessment of dental and periodontal status parameters are consistent with previously published studies, ensuring appropriate internal validity for our results.

## 5. Conclusions

The present single-institute case-control observational study provided information on the dental and periodontal status of AD patients vs. controls, demonstrating the substantial treatment need in individuals affected by the disease in Hungary and likely in countries with similar healthcare systems and epidemiological circumstances. In addition, while sex and common lifestyle correlate did not substantially alter the oral health of our participants, which corresponds to earlier studies in the field, smoking and alcohol use have demonstrated detrimental effects on periodontal health, which is increasingly understood to be a major contributor to AD pathogenesis. Dentists should be aware of the major symptoms and characteristics of each stage of illness and its pharmacological treatment (including adverse effects), understand the limitations of patients, and tacitly but assertively involve the patient’s caregivers, if available. To ensure the overarching goal of ensuring the availability of oral care for all AD patients, special attention (including funding and willing human resources) towards institutionalized patients and individuals residing in nursing homes is necessary.

## Figures and Tables

**Table 1:** Demographic characteristics and anamestic data of AD patients and controls.

	AD Patients		Controls	
	*n*	%	*n*	%
Gender				
Male	17	41.5%	17	41.5%
Female	24	58.5%	24	58.5%
Age				
66–77 years	11	26.8	11	26.8
78–87 years	17	41.5	17	41.5
88–97 years	13	31.7	13	31.7
Cigarettes/day				
0	23	56.1	19	46.3
1–4	13	31.7	16	39.0
≥5	5	12.3	6	14.7
Alcohol consumption (units/week)				
0	16	39.0	16	39.0
1–3	17	41.5	16	39.0
4–10	8	19.5	9	22.0
Coffee consumption (cups/day)				
0	8	19.5	11	26.8
1	21	51.2	25	60.9
≥2	12	29.3	5	12.3
Takes vitamin supplements				
No	8	19.5	26	63.4
Yes	33	80.5	15	36.6

AD: Alzheimer’s disease.

**Table 2:** Dental status parameters of AD patients and controls.

**AD Patients**					
	**D**	**M**	**F**	**DMF-T**	**DMF-S**
Total	4.56 ± 4.18	20.66 ± 7.29	1.02 ± 2.23	26.24 ± 3.58	119.83 ± 16.78
Gender					
Male	4.41 ± 3.59	19.53 ± 7.69	1.94 ± 3.05	25.88 ± 4.08	117.88 ± 18.19
Female	4.67 ± 4.65	21.45 ± 7.05	0.38 ± 1.5	26.50 ± 3.26	121.21 ± 15.96
**Controls**					
	**D**	**M**	**F**	**DMF-T**	**DMF-S**
Total	4.02 ± 4.95	21.37 ± 7.76	0.78 ± 1.82	26.15 ± 4.29	119.00 ± 18.93
Gender					
Male	5.47 ± 5.74	18.53 ± 9.41	0.52 ± 1.06	24.53 ± 5.91	112.35 ± 26.14
Female	3.00 ± 4.18	23.38 ± 5.74	0.95 ± 2.23	27.29 ± 2.12	123.71 ± 9.61

AD: Alzheimer’s disease; D: decayed; M: missing; F: filled; DMF-T: decayed, missing, and filled teeth; DMF-S: decayed, missing, and filled surfaces.

**Table 3:** Periodontal status parameters of AD patients and controls.

**AD Patients**				
	**Plaque Index (%)**	**BOP%**	**PD**	**AL**
Total	59.06 ± 15.45	62.65 ± 12.00	2.63 ± 0.56	2.85 ± 0.79
Gender				
Male	58.44 ± 17.39	65.44 ± 12.70	2.60 ± 0.73	2.73 ± 0.97
Female	59.75 ± 14.11	59.50 ± 11.11	2.68 ± 0.32	2.98 ± 0.52
**Controls**				
	**Plaque Index (%)**	**BOP%**	**PD**	**AL**
Total	41.35 ± 7.97	40.12 ± 10.86	2.29 ± 0.13	2.39 ± 0.41
Gender				
Male	43.22 ± 9.23	40.33 ± 13.22	2.27 ± 0.12	2.24 ± 0.73
Female	39.25 ± 6.21	39.88 ± 8.42	2.32 ± 0.15	2.53 ± 0.46

AD: Alzheimer’s disease; BOP%: bleeding on probing; PD: pocket depth; AL: attachment loss.

## Data Availability

All data generated during the study are presented in this paper.
